# Diaqua­bis(norfloxacinato)manganese(II) 2,2′-bipyridine solvate tetra­hydrate

**DOI:** 10.1107/S1600536809022831

**Published:** 2009-06-20

**Authors:** Yan-Jun Wang, Qiu-Yue Lin, Jie Feng, Na Wang

**Affiliations:** a Zhejiang Key Laboratory for Reactive Chemistry on Solid Surfaces, Institute of Physical Chemistry, Zhejiang Normal University, Jinhua, Zhejiang 321004, People’s Republic of China, and, College of Chemistry and Life Science, Zhejiang Normal University, Jinhua 321004, Zhejiang, People’s Republic of China

## Abstract

In the crystal structure of the title compound {systematic name: diaqua­bis[1-ethyl-6-fluoro-4-oxo-7-(piperazin-1-yl)-1,4-dihydro­quinoline-3-carboxyl­ato]manganese(II) 2,2′-bipyridine solvate tetra­hydrate}, [Mn(C_16_H_17_FN_3_O_3_)_2_(H_2_O)_2_]·C_10_H_8_N_2_·4H_2_O, the pyridone O atom and one carboxyl­ate O atom of the two norfloxacin ligands are bound to the Mn^II^ ion, which is located on an inversion centre, and occupy equatorial positions, while two aqua O atoms lie in apical positions, resulting in a distorted octa­hedral geometry. The crystal packing is stabilized by N—H⋯O and O—H⋯O hydrogen-bonding interactions.

## Related literature

For background, see: Dukhande *et al.* (2006 ▶).
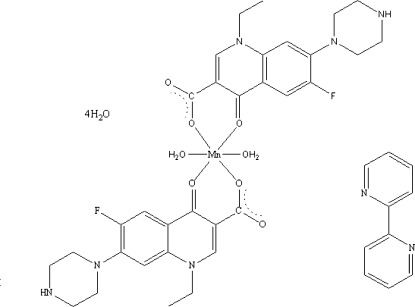


## Experimental

### 

#### Crystal data

[Mn(C_16_H_17_FN_3_O_3_)_2_(H_2_O)_2_]·C_10_H_8_N_2_·4H_2_O
*M* *_r_* = 955.87
Triclinic, 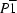

*a* = 9.5179 (4) Å
*b* = 11.4645 (2) Å
*c* = 11.6617 (2) Å
α = 118.8440 (10)°
β = 93.398 (2)°
γ = 97.258 (2)°
*V* = 1095.06 (5) Å^3^
*Z* = 1
Mo *K*α radiation
μ = 0.38 mm^−1^
*T* = 296 K
0.38 × 0.18 × 0.05 mm

#### Data collection

Bruker APEXII CCD area-detector diffractometer
Absorption correction: multi-scan (*SADABS*; Sheldrick, 1996 ▶) *T*_min_ = 0.921, *T*_max_ = 0.981
13676 measured reflections
3856 independent reflections
3208 reflections with *I* > 2σ(*I*)
*R*_int_ = 0.033

#### Refinement

*R*[*F* ^2^ > 2σ(*F* ^2^)] = 0.060
*wR*(*F* ^2^) = 0.199
*S* = 1.07
3856 reflections
310 parameters
9 restraints
H atoms treated by a mixture of independent and constrained refinement
Δρ_max_ = 1.14 e Å^−3^
Δρ_min_ = −0.51 e Å^−3^

### 

Data collection: *APEX2* (Bruker, 2004 ▶); cell refinement: *SAINT* (Bruker, 2004 ▶); data reduction: *SAINT*; program(s) used to solve structure: *SHELXS97* (Sheldrick, 2008 ▶); program(s) used to refine structure: *SHELXL97* (Sheldrick, 2008 ▶); molecular graphics: *SHELXTL* (Sheldrick, 2008 ▶); software used to prepare material for publication: *SHELXL97*.

## Supplementary Material

Crystal structure: contains datablocks I, global. DOI: 10.1107/S1600536809022831/at2812sup1.cif

Structure factors: contains datablocks I. DOI: 10.1107/S1600536809022831/at2812Isup2.hkl

Additional supplementary materials:  crystallographic information; 3D view; checkCIF report

## Figures and Tables

**Table 1:** Hydrogen-bond geometry (Å, °)

*D*—H⋯*A*	*D*—H	H⋯*A*	*D*⋯*A*	*D*—H⋯*A*
N3—H3*A*⋯O3^i^	0.86	2.23	2.725 (4)	117
N3—H3*A*⋯O3*W*	0.86	2.54	2.992 (4)	114
O2*W*—H2*WB*⋯O2*W*^ii^	0.85	1.97	2.789 (5)	163
O3*W*—H3*WA*⋯O3*W*^iii^	0.784 (19)	2.03 (2)	2.781 (6)	162 (6)
O3*W*—H3*WB*⋯N3	0.754 (19)	2.32 (4)	2.992 (4)	149 (5)
O1*W*—H1*WA*⋯N4	0.863 (19)	1.96 (2)	2.813 (4)	168 (5)
O1*W*—H1*WB*⋯O2*W*	0.842 (19)	2.24 (3)	3.050 (4)	162 (5)
O2*W*—H2*WA*⋯O1*W*	0.730 (17)	2.65 (4)	3.050 (4)	117 (4)

Symmetry codes: (i) 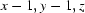; (ii) 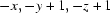; (iii) 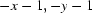, 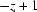.

**Fig. 1.**
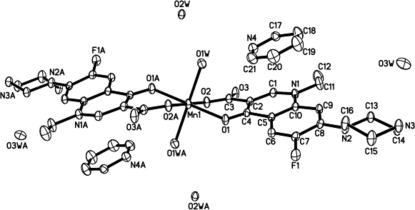
A view of the title molecule of (I) showing the atom-labelling scheme with displacement ellipsoids drawn at the 30% probability [symmetry code: (A) -x, -y, -z].

## supplementary crystallographic information

### Comment

1-Ethyl-6-fluoro-1,4-dihydro-4-oxo-7-(1-piperazinyl)-3-quinoline carboxylic acid (norfloxacin), is the third generation quinolone antibacterial drug with broad-spectrum antibacterial activity, especially for gram-negative bacteria. It can interfere with the synthesis of DNA, destroy the fission of cells in order to sterilize by inhibiting DNA gyrase. Manganese is an important trace element needed for normal physiological functions and development. It is also a cofactor or required metal ion for many enzymes, such as superoxide dismutase, glutamine synthetase and arginase (Dukhande *et al.*, 2006). Synthesis, characterization and biological activity studies of the manganese complexes have become one of the most attractive research fields in modern bioinorganic chemistry.

In the title compound, the Mn(II) ion in a inversion centre is coordinated with four oxygen atoms of the norfloxacin ligands in the equatorial positions while two oxygen atoms of the water occupy the axial positions resulting in a distorted octahedral geometry around the central metal atom. The Mn—O bond distances arising from the two carbonyl oxygen atoms O1 are longer, [2.157 (2) Å], than those arising from the carboxylate oxygen atoms O2 [2.132 (2) Å]. The axial average linkages between manganese and oxygen atoms of water are substantially longer [2.212 (3) Å] than the equatorial bond distances. The bond angles O1—Mn1—O1A, O2—Mn1—O2A and O1W—Mn1—O1WA are 180° while the bond angles O2—Mn1—O1 and O2A—Mn1—O1 open up slightly from 82.73 (9)° to 97.27 (9)°, resulting in a slight distortion from the idealized octahedral geometry.

The crystal packing is stabilized by N—H···O and O—H···O hydrogen bonding interactions (Table 1).

### Experimental

A mixture of 0.1 mmol norfloxacin, 0.1 mmol MnCl_2_4H_2_O, 0.1 mmol 2,2'-bipyridine and 10 mL distilled water was sealed in a 25 mL Teflon-lined stainless vessel and heated at 433 K for 3 d, then cooled slowly to room temperature. The solution was filtered and block yellow crystals were obtained.

### Refinement

The H atoms bonded to C atoms were positioned geometrically and refined using a riding model [aromatic C—H = 0.93 Å, aliphatic C—H = 0.97 Å and N—H = 0.86 Å, *U*_iso_(H) = 1.2*U*_eq_(C),]. The H atoms bonded to O atoms were located in a difference Fourier maps and refined with O—H distance restraints of 0.85 (2) and *U*_iso_(H) = 1.5*U*_eq_(O).

### Crystal data

**Table:**

[Mn(C_16_H_17_FN_3_O_3_)_2_(H_2_O)_2_]·C_10_H_8_N_2_·4H_2_O	*Z* = 1
*M**_r_* = 955.87	*F*(000) = 501
Triclinic, *P*1	*D*_x_ = 1.449 Mg m^−^^3^
Hall symbol: -P 1	Mo *K*α radiation, λ = 0.71073 Å
*a* = 9.5179 (4) Å	Cell parameters from 3687 reflections
*b* = 11.4645 (2) Å	θ = 2.0–25.0°
*c* = 11.6617 (2) Å	µ = 0.38 mm^−^^1^
α = 118.844 (1)°	*T* = 296 K
β = 93.398 (2)°	Block, yellow
γ = 97.258 (2)°	0.38 × 0.18 × 0.05 mm
*V* = 1095.06 (5) Å^3^	

### Data collection

**Table:**

Bruker APEXII CCD area-detector diffractometer	3856 independent reflections
Radiation source: fine-focus sealed tube	3208 reflections with *I* > 2σ(*I*)
graphite	*R*_int_ = 0.033
ω scans	θ_max_ = 25.0°, θ_min_ = 2.0°
Absorption correction: multi-scan (*SADABS*; Sheldrick, 1996)	*h* = −11→11
*T*_min_ = 0.921, *T*_max_ = 0.981	*k* = −13→13
13676 measured reflections	*l* = −13→13

### Refinement

**Table:**

Refinement on *F*^2^	Primary atom site location: structure-invariant direct methods
Least-squares matrix: full	Secondary atom site location: difference Fourier map
*R*[*F*^2^ > 2σ(*F*^2^)] = 0.060	Hydrogen site location: inferred from neighbouring sites
*wR*(*F*^2^) = 0.199	H atoms treated by a mixture of independent and constrained refinement
*S* = 1.07	*w* = 1/[σ^2^(*F*_o_^2^) + (0.1291*P*)^2^ + 0.9928*P*] where *P* = (*F*_o_^2^ + 2*F*_c_^2^)/3
3856 reflections	(Δ/σ)_max_ < 0.001
310 parameters	Δρ_max_ = 1.14 e Å^−^^3^
9 restraints	Δρ_min_ = −0.51 e Å^−^^3^

### Special details

**Table:**

Geometry. All e.s.d.'s (except the e.s.d. in the dihedral angle between two l.s. planes) are estimated using the full covariance matrix. The cell e.s.d.'s are taken into account individually in the estimation of e.s.d.'s in distances, angles and torsion angles; correlations between e.s.d.'s in cell parameters are only used when they are defined by crystal symmetry. An approximate (isotropic) treatment of cell e.s.d.'s is used for estimating e.s.d.'s involving l.s. planes.
Refinement. Refinement of *F*^2^ against ALL reflections. The weighted *R*-factor *wR* and goodness of fit *S* are based on *F*^2^, conventional *R*-factors *R* are based on *F*, with *F* set to zero for negative *F*^2^. The threshold expression of *F*^2^ > σ(*F*^2^) is used only for calculating *R*-factors(gt) *etc*. and is not relevant to the choice of reflections for refinement. *R*-factors based on *F*^2^ are statistically about twice as large as those based on *F*, and *R*- factors based on ALL data will be even larger.

### Fractional atomic coordinates and isotropic or equivalent isotropic displacement parameters (Å^2^)

**Table:**

	*x*	*y*	*z*	*U*_iso_*/*U*_eq_	
Mn1	0.0000	0.0000	0.0000	0.0298 (3)	
F1	−0.3877 (2)	−0.5950 (2)	−0.0804 (2)	0.0422 (5)	
N1	0.1045 (3)	−0.2919 (3)	0.2598 (3)	0.0304 (6)	
N2	−0.3216 (3)	−0.6391 (3)	0.1294 (3)	0.0317 (7)	
N3	−0.5036 (3)	−0.8056 (3)	0.2005 (3)	0.0387 (7)	
H3A	−0.5571	−0.8227	0.2494	0.046*	
N4	−0.1120 (3)	−0.0184 (3)	0.3595 (3)	0.0409 (7)	
O1W	−0.0371 (3)	0.1057 (3)	0.2090 (3)	0.0439 (7)	
H1WA	−0.047 (5)	0.071 (4)	0.260 (4)	0.066*	
H1WB	0.012 (5)	0.182 (3)	0.263 (4)	0.066*	
O1	−0.0540 (3)	−0.1938 (2)	−0.0106 (2)	0.0356 (6)	
O2	0.2086 (2)	−0.0111 (2)	0.0669 (3)	0.0363 (6)	
O2W	0.0743 (2)	0.3979 (2)	0.4232 (2)	0.0312 (5)	
H2WB	0.0155	0.4515	0.4561	0.037*	
H2WA	0.074 (4)	0.366 (4)	0.352 (2)	0.047*	
O3	0.3729 (3)	−0.0402 (3)	0.1876 (3)	0.0445 (7)	
O3W	−0.6092 (3)	−0.6120 (4)	0.4471 (3)	0.0510 (8)	
H3WA	−0.553 (5)	−0.548 (3)	0.462 (5)	0.076*	
H3WB	−0.610 (6)	−0.678 (3)	0.385 (4)	0.076*	
C1	0.1824 (3)	−0.2018 (3)	0.2366 (3)	0.0294 (7)	
H1A	0.2717	−0.1597	0.2870	0.035*	
C2	0.1411 (3)	−0.1664 (3)	0.1442 (3)	0.0277 (7)	
C3	0.2478 (3)	−0.0647 (3)	0.1329 (3)	0.0304 (7)	
C4	0.0043 (3)	−0.2260 (3)	0.0674 (3)	0.0264 (7)	
C5	−0.0729 (3)	−0.3357 (3)	0.0820 (3)	0.0265 (7)	
C6	−0.1985 (3)	−0.4144 (3)	−0.0032 (3)	0.0291 (7)	
H6A	−0.2307	−0.3989	−0.0707	0.035*	
C7	−0.2736 (3)	−0.5133 (3)	0.0123 (3)	0.0296 (7)	
C8	−0.2372 (3)	−0.5373 (3)	0.1170 (3)	0.0283 (7)	
C9	−0.1118 (3)	−0.4615 (3)	0.1998 (3)	0.0297 (7)	
H9A	−0.0830	−0.4749	0.2696	0.036*	
C10	−0.0263 (3)	−0.3640 (3)	0.1805 (3)	0.0268 (7)	
C11	0.1577 (4)	−0.3222 (5)	0.3635 (4)	0.0483 (10)	
H11A	0.1015	−0.2850	0.4357	0.058*	
H11B	0.1408	−0.4196	0.3266	0.058*	
C12	0.3105 (5)	−0.2698 (6)	0.4181 (5)	0.0667 (14)	
H12A	0.3339	−0.2931	0.4848	0.100*	
H12B	0.3289	−0.1732	0.4563	0.100*	
H12C	0.3679	−0.3091	0.3486	0.100*	
C13	−0.2658 (4)	−0.6694 (3)	0.2297 (4)	0.0333 (8)	
H13A	−0.2753	−0.5977	0.3168	0.040*	
H13B	−0.1650	−0.6741	0.2256	0.040*	
C14	−0.3468 (4)	−0.8034 (4)	0.2073 (4)	0.0348 (8)	
H14A	−0.3265	−0.8764	0.1255	0.042*	
H14B	−0.3142	−0.8182	0.2789	0.042*	
C15	−0.5550 (4)	−0.7743 (4)	0.0974 (5)	0.0480 (10)	
H15A	−0.6568	−0.7733	0.0955	0.058*	
H15B	−0.5386	−0.8432	0.0113	0.058*	
C16	−0.4754 (4)	−0.6377 (4)	0.1281 (5)	0.0428 (10)	
H16A	−0.5095	−0.6158	0.0620	0.051*	
H16B	−0.4933	−0.5688	0.2136	0.051*	
C17	−0.0729 (4)	−0.0353 (4)	0.4622 (3)	0.0361 (8)	
C18	−0.1623 (5)	−0.1148 (5)	0.4961 (5)	0.0542 (11)	
H18A	−0.1329	−0.1265	0.5668	0.065*	
C19	−0.2967 (5)	−0.1769 (6)	0.4233 (5)	0.0672 (14)	
H19A	−0.3587	−0.2305	0.4449	0.081*	
C20	−0.3377 (5)	−0.1586 (5)	0.3188 (5)	0.0598 (12)	
H20A	−0.4278	−0.1978	0.2692	0.072*	
C21	−0.2410 (4)	−0.0807 (4)	0.2904 (4)	0.0492 (10)	
H21A	−0.2671	−0.0705	0.2181	0.059*	

### Atomic displacement parameters (Å^2^)

**Table:**

	*U*^11^	*U*^22^	*U*^33^	*U*^12^	*U*^13^	*U*^23^
Mn1	0.0279 (4)	0.0306 (4)	0.0384 (5)	−0.0017 (3)	0.0012 (3)	0.0249 (3)
F1	0.0336 (11)	0.0393 (11)	0.0501 (13)	−0.0151 (9)	−0.0137 (9)	0.0265 (10)
N1	0.0239 (14)	0.0362 (15)	0.0370 (16)	−0.0042 (11)	−0.0018 (12)	0.0255 (13)
N2	0.0214 (14)	0.0323 (15)	0.0502 (18)	−0.0029 (11)	0.0024 (12)	0.0291 (14)
N3	0.0256 (14)	0.0447 (18)	0.060 (2)	−0.0028 (13)	0.0082 (14)	0.0385 (16)
N4	0.0401 (18)	0.0442 (18)	0.0402 (17)	0.0010 (14)	0.0012 (14)	0.0241 (15)
O1W	0.0558 (17)	0.0430 (15)	0.0369 (14)	0.0043 (13)	0.0047 (13)	0.0242 (12)
O1	0.0354 (13)	0.0339 (13)	0.0440 (14)	−0.0065 (10)	−0.0067 (11)	0.0286 (12)
O2	0.0286 (12)	0.0425 (14)	0.0511 (15)	−0.0023 (10)	0.0017 (11)	0.0360 (13)
O2W	0.0290 (12)	0.0291 (12)	0.0373 (13)	−0.0011 (9)	−0.0061 (10)	0.0205 (11)
O3	0.0266 (13)	0.0507 (16)	0.0700 (19)	−0.0133 (11)	−0.0098 (12)	0.0470 (15)
O3W	0.0297 (14)	0.072 (2)	0.0455 (17)	0.0075 (14)	0.0191 (13)	0.0240 (15)
C1	0.0230 (15)	0.0297 (17)	0.0387 (18)	−0.0024 (13)	0.0007 (13)	0.0214 (15)
C2	0.0228 (15)	0.0278 (16)	0.0358 (18)	0.0001 (13)	0.0041 (13)	0.0193 (14)
C3	0.0263 (17)	0.0306 (17)	0.0378 (19)	−0.0021 (13)	0.0033 (14)	0.0215 (15)
C4	0.0276 (16)	0.0245 (16)	0.0314 (17)	0.0023 (13)	0.0056 (13)	0.0176 (14)
C5	0.0237 (15)	0.0254 (16)	0.0338 (17)	0.0010 (12)	0.0032 (13)	0.0180 (14)
C6	0.0281 (17)	0.0302 (17)	0.0329 (17)	0.0017 (13)	0.0010 (14)	0.0199 (14)
C7	0.0240 (16)	0.0270 (16)	0.0363 (18)	−0.0027 (13)	−0.0012 (13)	0.0168 (14)
C8	0.0263 (16)	0.0249 (16)	0.0392 (19)	0.0013 (13)	0.0054 (14)	0.0207 (15)
C9	0.0273 (16)	0.0314 (17)	0.0364 (18)	−0.0009 (13)	0.0008 (14)	0.0232 (15)
C10	0.0238 (16)	0.0266 (16)	0.0329 (17)	0.0011 (13)	0.0027 (13)	0.0179 (14)
C11	0.036 (2)	0.069 (3)	0.058 (3)	−0.0065 (18)	−0.0070 (18)	0.051 (2)
C12	0.056 (3)	0.090 (4)	0.069 (3)	−0.001 (3)	−0.007 (2)	0.055 (3)
C13	0.0281 (17)	0.0341 (18)	0.044 (2)	−0.0046 (14)	−0.0002 (15)	0.0273 (16)
C14	0.0279 (17)	0.0366 (19)	0.049 (2)	0.0003 (14)	0.0070 (15)	0.0292 (17)
C15	0.0270 (18)	0.055 (2)	0.075 (3)	−0.0097 (17)	−0.0036 (18)	0.047 (2)
C16	0.0243 (17)	0.047 (2)	0.075 (3)	0.0002 (15)	0.0061 (17)	0.046 (2)
C17	0.0365 (19)	0.0367 (19)	0.0349 (19)	0.0026 (15)	0.0036 (16)	0.0186 (16)
C18	0.045 (2)	0.071 (3)	0.057 (3)	−0.008 (2)	−0.001 (2)	0.045 (2)
C19	0.048 (3)	0.084 (4)	0.076 (3)	−0.020 (2)	−0.004 (2)	0.053 (3)
C20	0.043 (2)	0.070 (3)	0.064 (3)	−0.006 (2)	−0.006 (2)	0.035 (3)
C21	0.046 (2)	0.058 (3)	0.042 (2)	0.0024 (19)	−0.0021 (18)	0.027 (2)

### Geometric parameters (Å, °)

**Table:**

Mn1—O2	2.132 (2)	C5—C10	1.400 (5)
Mn1—O2^i^	2.132 (2)	C6—C7	1.356 (5)
Mn1—O1^i^	2.157 (2)	C6—H6A	0.9300
Mn1—O1	2.157 (2)	C7—C8	1.408 (5)
Mn1—O1W^i^	2.212 (3)	C8—C9	1.380 (5)
Mn1—O1W	2.212 (3)	C9—C10	1.414 (4)
F1—C7	1.361 (4)	C9—H9A	0.9300
N1—C1	1.338 (4)	C11—C12	1.477 (6)
N1—C10	1.398 (4)	C11—H11A	0.9700
N1—C11	1.488 (4)	C11—H11B	0.9700
N2—C8	1.403 (4)	C12—H12A	0.9600
N2—C13	1.462 (4)	C12—H12B	0.9600
N2—C16	1.465 (4)	C12—H12C	0.9600
N3—C15	1.486 (5)	C13—C14	1.520 (4)
N3—C14	1.486 (4)	C13—H13A	0.9700
N3—H3A	0.8600	C13—H13B	0.9700
N4—C21	1.332 (5)	C14—H14A	0.9700
N4—C17	1.342 (5)	C14—H14B	0.9700
O1W—H1WA	0.86 (5)	C15—C16	1.510 (5)
O1W—H1WB	0.84 (4)	C15—H15A	0.9700
O1—C4	1.260 (4)	C15—H15B	0.9700
O2—C3	1.261 (4)	C16—H16A	0.9700
O2W—H2WB	0.8500	C16—H16B	0.9700
O2W—H2WA	0.730 (17)	C17—C18	1.380 (6)
O3—C3	1.248 (4)	C17—C17^ii^	1.497 (7)
O3W—H3WA	0.79 (5)	C18—C19	1.386 (6)
O3W—H3WB	0.75 (4)	C18—H18A	0.9300
C1—C2	1.376 (5)	C19—C20	1.376 (7)
C1—H1A	0.9300	C19—H19A	0.9300
C2—C4	1.418 (4)	C20—C21	1.366 (6)
C2—C3	1.508 (4)	C20—H20A	0.9300
C4—C5	1.463 (4)	C21—H21A	0.9300
C5—C6	1.397 (4)		
			
O2—Mn1—O2^i^	180.00 (14)	C8—C9—H9A	119.4
O2—Mn1—O1^i^	97.27 (9)	C10—C9—H9A	119.4
O2^i^—Mn1—O1^i^	82.73 (9)	N1—C10—C5	118.4 (3)
O2—Mn1—O1	82.73 (9)	N1—C10—C9	121.4 (3)
O2^i^—Mn1—O1	97.27 (9)	C5—C10—C9	120.2 (3)
O1^i^—Mn1—O1	180.00 (18)	C12—C11—N1	115.8 (3)
O2—Mn1—O1W^i^	91.93 (10)	C12—C11—H11A	108.3
O2^i^—Mn1—O1W^i^	88.07 (10)	N1—C11—H11A	108.3
O1^i^—Mn1—O1W^i^	90.90 (10)	C12—C11—H11B	108.3
O1—Mn1—O1W^i^	89.10 (10)	N1—C11—H11B	108.3
O2—Mn1—O1W	88.07 (10)	H11A—C11—H11B	107.4
O2^i^—Mn1—O1W	91.93 (10)	C11—C12—H12A	109.5
O1^i^—Mn1—O1W	89.10 (10)	C11—C12—H12B	109.5
O1—Mn1—O1W	90.90 (10)	H12A—C12—H12B	109.5
O1W^i^—Mn1—O1W	180.00 (16)	C11—C12—H12C	109.5
C1—N1—C10	119.2 (3)	H12A—C12—H12C	109.5
C1—N1—C11	121.4 (3)	H12B—C12—H12C	109.5
C10—N1—C11	119.4 (3)	N2—C13—C14	110.3 (3)
C8—N2—C13	116.8 (3)	N2—C13—H13A	109.6
C8—N2—C16	117.3 (3)	C14—C13—H13A	109.6
C13—N2—C16	111.3 (3)	N2—C13—H13B	109.6
C15—N3—C14	110.4 (3)	C14—C13—H13B	109.6
C15—N3—H3A	124.8	H13A—C13—H13B	108.1
C14—N3—H3A	124.8	N3—C14—C13	111.6 (3)
C21—N4—C17	117.9 (3)	N3—C14—H14A	109.3
Mn1—O1W—H1WA	126 (3)	C13—C14—H14A	109.3
Mn1—O1W—H1WB	121 (3)	N3—C14—H14B	109.3
H1WA—O1W—H1WB	100 (3)	C13—C14—H14B	109.3
C4—O1—Mn1	124.5 (2)	H14A—C14—H14B	108.0
C3—O2—Mn1	130.6 (2)	N3—C15—C16	109.1 (3)
H2WB—O2W—H2WA	117.1	N3—C15—H15A	109.9
H3WA—O3W—H3WB	120 (4)	C16—C15—H15A	109.9
N1—C1—C2	125.3 (3)	N3—C15—H15B	109.9
N1—C1—H1A	117.3	C16—C15—H15B	109.9
C2—C1—H1A	117.3	H15A—C15—H15B	108.3
C1—C2—C4	119.1 (3)	N2—C16—C15	110.1 (3)
C1—C2—C3	116.2 (3)	N2—C16—H16A	109.6
C4—C2—C3	124.7 (3)	C15—C16—H16A	109.6
O3—C3—O2	123.0 (3)	N2—C16—H16B	109.6
O3—C3—C2	117.6 (3)	C15—C16—H16B	109.6
O2—C3—C2	119.3 (3)	H16A—C16—H16B	108.2
O1—C4—C2	126.3 (3)	N4—C17—C18	121.7 (4)
O1—C4—C5	118.6 (3)	N4—C17—C17^ii^	116.9 (4)
C2—C4—C5	115.1 (3)	C18—C17—C17^ii^	121.5 (4)
C6—C5—C10	118.3 (3)	C17—C18—C19	119.0 (4)
C6—C5—C4	119.6 (3)	C17—C18—H18A	120.5
C10—C5—C4	122.1 (3)	C19—C18—H18A	120.5
C7—C6—C5	120.1 (3)	C20—C19—C18	119.5 (4)
C7—C6—H6A	119.9	C20—C19—H19A	120.2
C5—C6—H6A	119.9	C18—C19—H19A	120.2
C6—C7—F1	117.7 (3)	C21—C20—C19	117.5 (4)
C6—C7—C8	123.4 (3)	C21—C20—H20A	121.2
F1—C7—C8	118.9 (3)	C19—C20—H20A	121.2
C9—C8—N2	122.8 (3)	N4—C21—C20	124.4 (4)
C9—C8—C7	116.5 (3)	N4—C21—H21A	117.8
N2—C8—C7	120.5 (3)	C20—C21—H21A	117.8
C8—C9—C10	121.2 (3)		
			
O2—Mn1—O1—C4	33.6 (3)	C16—N2—C8—C7	−52.3 (5)
O2^i^—Mn1—O1—C4	−146.4 (3)	C6—C7—C8—C9	−5.4 (5)
O1W^i^—Mn1—O1—C4	125.6 (3)	F1—C7—C8—C9	173.0 (3)
O1W—Mn1—O1—C4	−54.4 (3)	C6—C7—C8—N2	178.3 (3)
O1^i^—Mn1—O2—C3	147.8 (3)	F1—C7—C8—N2	−3.3 (5)
O1—Mn1—O2—C3	−32.2 (3)	N2—C8—C9—C10	177.1 (3)
O1W^i^—Mn1—O2—C3	−121.0 (3)	C7—C8—C9—C10	0.8 (5)
O1W—Mn1—O2—C3	59.0 (3)	C1—N1—C10—C5	−2.0 (5)
C10—N1—C1—C2	4.2 (5)	C11—N1—C10—C5	−178.8 (3)
C11—N1—C1—C2	−179.2 (3)	C1—N1—C10—C9	179.2 (3)
N1—C1—C2—C4	1.4 (5)	C11—N1—C10—C9	2.5 (5)
N1—C1—C2—C3	−178.6 (3)	C6—C5—C10—N1	175.6 (3)
Mn1—O2—C3—O3	−165.0 (3)	C4—C5—C10—N1	−5.4 (5)
Mn1—O2—C3—C2	16.9 (5)	C6—C5—C10—C9	−5.7 (5)
C1—C2—C3—O3	13.4 (5)	C4—C5—C10—C9	173.4 (3)
C4—C2—C3—O3	−166.6 (3)	C8—C9—C10—N1	−176.7 (3)
C1—C2—C3—O2	−168.3 (3)	C8—C9—C10—C5	4.6 (5)
C4—C2—C3—O2	11.6 (5)	C1—N1—C11—C12	−12.3 (6)
Mn1—O1—C4—C2	−22.6 (5)	C10—N1—C11—C12	164.3 (4)
Mn1—O1—C4—C5	157.9 (2)	C8—N2—C13—C14	−165.1 (3)
C1—C2—C4—O1	172.2 (3)	C16—N2—C13—C14	56.5 (4)
C3—C2—C4—O1	−7.8 (5)	C15—N3—C14—C13	55.3 (4)
C1—C2—C4—C5	−8.3 (4)	N2—C13—C14—N3	−54.1 (4)
C3—C2—C4—C5	171.8 (3)	C14—N3—C15—C16	−57.8 (4)
O1—C4—C5—C6	9.1 (5)	C8—N2—C16—C15	161.5 (3)
C2—C4—C5—C6	−170.5 (3)	C13—N2—C16—C15	−60.3 (4)
O1—C4—C5—C10	−170.0 (3)	N3—C15—C16—N2	60.2 (4)
C2—C4—C5—C10	10.4 (5)	C21—N4—C17—C18	0.2 (6)
C10—C5—C6—C7	1.4 (5)	C21—N4—C17—C17^ii^	179.8 (4)
C4—C5—C6—C7	−177.7 (3)	N4—C17—C18—C19	−1.0 (7)
C5—C6—C7—F1	−174.1 (3)	C17^ii^—C17—C18—C19	179.4 (5)
C5—C6—C7—C8	4.3 (5)	C17—C18—C19—C20	0.3 (8)
C13—N2—C8—C9	−4.2 (5)	C18—C19—C20—C21	1.1 (8)
C16—N2—C8—C9	131.6 (4)	C17—N4—C21—C20	1.4 (7)
C13—N2—C8—C7	171.9 (3)	C19—C20—C21—N4	−2.0 (8)

Symmetry codes: (i) −*x*, −*y*, −*z*; (ii) −*x*, −*y*, −*z*+1.

### Hydrogen-bond geometry (Å, °)

**Table:**

*D*—H···*A*	*D*—H	H···*A*	*D*···*A*	*D*—H···*A*
N3—H3A···O3^iii^	0.86	2.23	2.725 (4)	117
N3—H3A···O3W	0.86	2.54	2.992 (4)	114
O2W—H2WB···O2W^iv^	0.85	1.97	2.789 (5)	163
O3W—H3WA···O3W^v^	0.78 (2)	2.03 (2)	2.781 (6)	162 (6)
O3W—H3WB···N3	0.75 (2)	2.32 (4)	2.992 (4)	149 (5)
O1W—H1WA···N4	0.86 (2)	1.96 (2)	2.813 (4)	168 (5)
O1W—H1WB···O2W	0.84 (2)	2.24 (3)	3.050 (4)	162 (5)
O2W—H2WA···O1W	0.73 (2)	2.65 (4)	3.050 (4)	117 (4)

Symmetry codes: (iii) *x*−1, *y*−1, *z*; (iv) −*x*, −*y*+1, −*z*+1; (v) −*x*−1, −*y*−1, −*z*+1.
